# Performance of Large Language Models (ChatGPT, Bing Search, and Google Bard) in Solving Case Vignettes in Physiology

**DOI:** 10.7759/cureus.42972

**Published:** 2023-08-04

**Authors:** Anup Kumar D Dhanvijay, Mohammed Jaffer Pinjar, Nitin Dhokane, Smita R Sorte, Amita Kumari, Himel Mondal

**Affiliations:** 1 Physiology, All India Institute of Medical Sciences, Deoghar, Deoghar, IND; 2 Physiology, Government Medical College, Sindhudurg, Oros, IND; 3 Physiology, All India Institute of Medical Sciences, Nagpur, Nagpur, IND

**Keywords:** problem-based learning, self-directed learning (sdl), medical education, case vignette, physiology, bing, bard, chatgpt, language model, artificial intelligence

## Abstract

Background

Large language models (LLMs) have emerged as powerful tools capable of processing and generating human-like text. These LLMs, such as ChatGPT (OpenAI Incorporated, Mission District, San Francisco, United States), Google Bard (Alphabet Inc., CA, US), and Microsoft Bing (Microsoft Corporation, WA, US), have been applied across various domains, demonstrating their potential to assist in solving complex tasks and improving information accessibility. However, their application in solving case vignettes in physiology has not been explored. This study aimed to assess the performance of three LLMs, namely, ChatGPT (3.5; free research version), Google Bard (Experiment), and Microsoft Bing (precise), in answering cases vignettes in Physiology.

Methods

This cross-sectional study was conducted in July 2023. A total of 77 case vignettes in physiology were prepared by two physiologists and were validated by two other content experts. These cases were presented to each LLM, and their responses were collected. Two physiologists independently rated the answers provided by the LLMs based on their accuracy. The ratings were measured on a scale from 0 to 4 according to the structure of the observed learning outcome (pre-structural = 0, uni-structural = 1, multi-structural = 2, relational = 3, extended-abstract). The scores among the LLMs were compared by Friedman’s test and inter-observer agreement was checked by the intraclass correlation coefficient (ICC).

Results

The overall scores for ChatGPT, Bing, and Bard in the study, with a total of 77 cases, were found to be 3.19±0.3, 2.15±0.6, and 2.91±0.5, respectively, p<0.0001. Hence, ChatGPT 3.5 (free version) obtained the highest score, Bing (Precise) had the lowest score, and Bard (Experiment) fell in between the two in terms of performance. The average ICC values for ChatGPT, Bing, and Bard were 0.858 (95% CI: 0.777 to 0.91, p<0.0001), 0.975 (95% CI: 0.961 to 0.984, p<0.0001), and 0.964 (95% CI: 0.944 to 0.977, p<0.0001), respectively.

Conclusion

ChatGPT outperformed Bard and Bing in answering case vignettes in physiology. Hence, students and teachers may think about choosing LLMs for their educational purposes accordingly for case-based learning in physiology. Further exploration of their capabilities is needed for adopting those in medical education and support for clinical decision-making.

## Introduction

With rapid advancements in natural language processing, large language models (LLMs) have emerged as powerful tools capable of processing and generating human-like text. These LLMs, such as ChatGPT (OpenAI Incorporated, Mission District, San Francisco, United States), Google Bard (Alphabet Inc., CA, US), and Microsoft Bing (Microsoft Corporation, WA, US), have been applied across various domains, demonstrating their potential to assist in solving complex tasks and improving information accessibility [1]. In the context of medical education and practice, the ability of LLMs to provide accurate and contextually relevant responses holds significant promise [2]. There are various domains of medical education like solving higher-order problems, generating questions, and preparing content for PowerPoint slides where LLMs can help teachers and students [3]. However, their application in solving case vignettes in physiology remains relatively unexplored.

The background of this study is rooted in the growing interest in leveraging LLMs to enhance medical education and support clinical decision-making. Traditional medical teaching methodologies, including case-based learning, have long been utilized to facilitate critical thinking and problem-solving skills in medical students [4]. The integration of LLMs into this educational framework offers an opportunity to explore novel approaches to learning and problem-solving in the medical field.

The potential implications of this study are twofold. Firstly, it seeks to shed light on the performance of three prominent LLMs, namely, ChatGPT-3.5 (free research version), Google Bard, and Microsoft Bing, in answering case vignettes in the domain of physiology. Understanding how these LLMs perform in this specific context can inform educators and practitioners about their efficacy as supplementary tools in medical education [5]. Secondly, the study's findings could have broader implications for the integration of LLMs into medical practice. If LLMs demonstrate proficiency in accurately answering case vignettes, they may be employed in various medical settings to support clinical decision-making, provide quick access to relevant medical information, and potentially reduce the workload of healthcare professionals [6]. Nevertheless, it is essential to critically assess the reliability and limitations of LLMs, as their responses can be influenced by the data they were trained on, leading to potential biases or inaccuracies.

## Materials and methods

Type and settings

This was a cross-sectional observational study involving data audit sourced from three public domain LLMs. The study was conducted as a comparative analysis of LLMs in solving cases vignettes in the domain of physiology. The research was carried out in an academic setting, involving the collaboration of two physiologists as raters. The three LLMs under investigation were ChatGPT, Bard, and Bing.

Preparation of case vignettes

**Figure 1:**
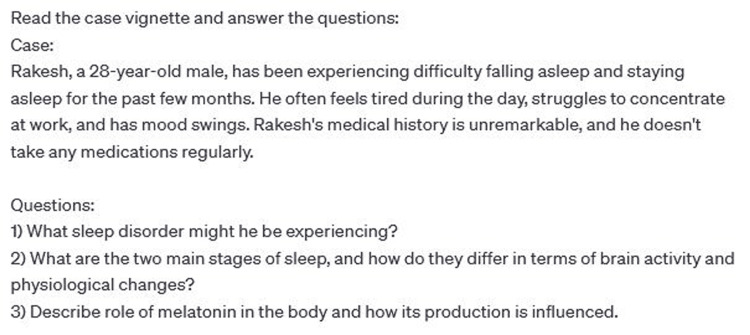
An example of a case vignette and related questions

A total of 77 case vignettes were carefully curated to encompass a diverse range of physiological and pathophysiological scenarios. However, the questions were set for the level an undergraduate student with knowledge of physiology can answer it. An example is shown in Figure 1.

Each case vignette was designed to present challenging medical situations, requiring critical thinking and expertise in physiology for accurate and contextually appropriate responses. Two physiologists created the case vignettes which were reviewed and validated by another two experts to ensure their relevance and accuracy.

Data collection method

For data collection, the selected case vignettes were presented individually to each of the three LLMs - ChatGPT 3.5 (free research version), Google Bard (Alphabet Inc., CA, US), and Bing Chat (Precise search) (Microsoft Corporation, WA, US). The LLMs were given access to the case vignettes and asked to provide written responses to each scenario with a prompt - "Read the case vignette and answer the questions". These responses were then compiled and stored for further analysis.

Rating of answers

**Figure 2:**
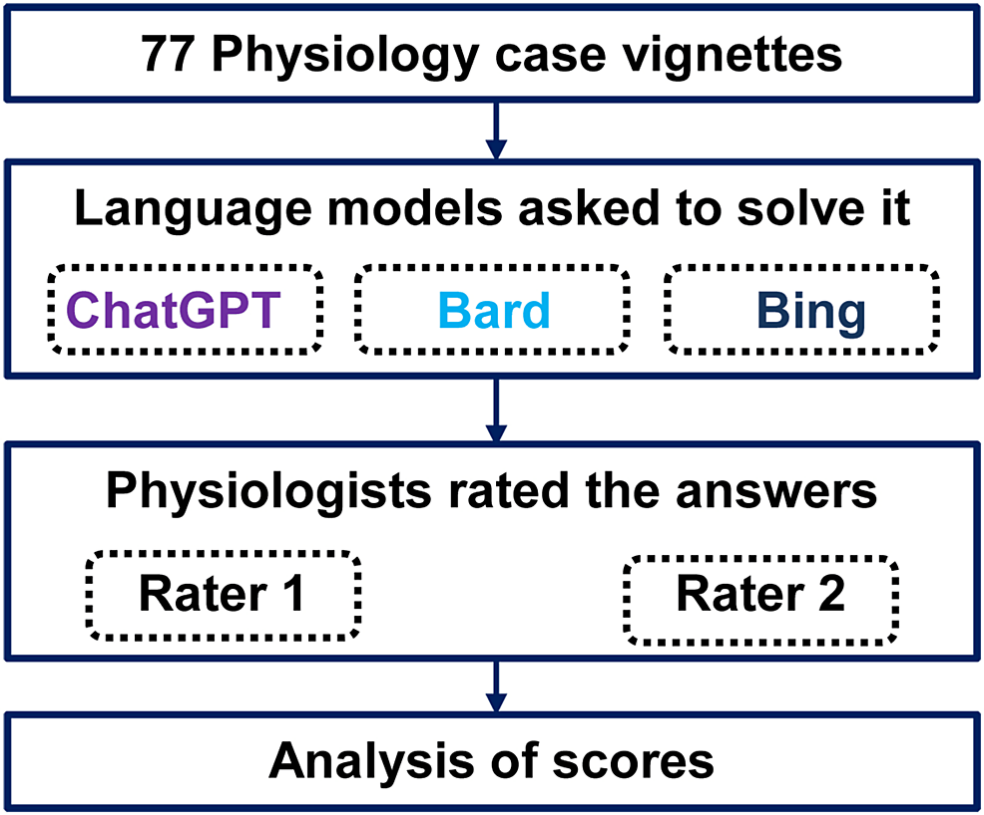
Brief study process flow chart

Two experienced physiologists independently rated the responses generated by the LLMs based on accuracy and appropriateness. The ratings were performed on a numerical scale from 0 to 4, with higher scores indicating more accurate and contextually relevant answers. This rating was according to the structure of observed learning outcome and scored as pre-structural = 0, uni-structural = 1, multi-structural = 2, relational = 3, extended-abstract = 4. The study process in a flow chart is shown in Figure 2.

Statistical analysis

The obtained ratings from the two physiologists for each LLM's responses were subjected to statistical analysis. Mean and standard deviation were calculated for each LLM's scores to assess their overall performance in solving the cases vignettes. Friedman’s test was applied to compare the variances among the scores. Inter-observer agreement was checked by the intraclass correlation coefficient (ICC). We used IBM SPSS Statistics for Windows, Version 20 (Released 2011; IBM Corp., Armonk, New York, United States) for conducting statistical tests, and a p-value <0.05 was considered statistically significant.

Ethics

The study adhered to ethical guidelines for research. There were no human research participants in this study. The data of case vignettes were fictitious and any resemblance with any subject or patient is coincidental. Hence, this study does not need ethical clearance according to the Indian Council of Medical Research (ICMR)’s comprehensive ethics guidelines for conducting research involving human subjects.

## Results

**Figure 3:**
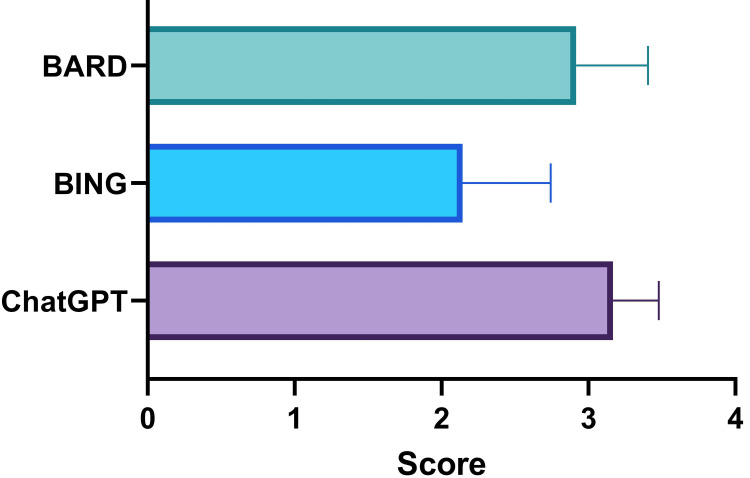
Scores of ChatGPT, Bing, and Bard in solving cases in physiology as rated by the first rater

A total of 77 cases in physiology were analyzed by two physiologists. The scores obtained by ChatGPT, Bing, and Bard were 3.17±0.31, 2.14±0.6, and 2.92±0.49, respectively as rated by the first rater as shown in Figure 3. There was a statistically significant (p<0.0001) difference among the scores with the highest score obtained by ChatGPT and the lowest by Bing.

**Figure 4:**
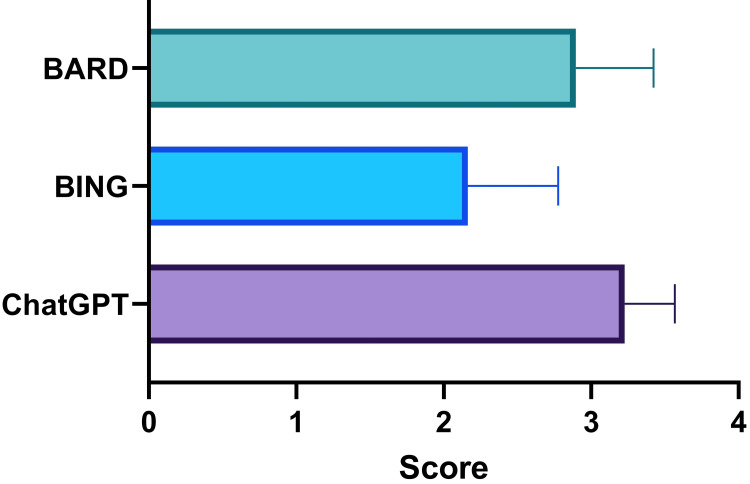
Scores of ChatGPT, Bing, and Bard in solving cases in physiology as rated by the second rater

A similar pattern is seen in the score rated by the second rater as shown in Figure 4. The scores were 3.23±0.34, 2.16±0.61, and 2.89±0.53, respectively, p<0.0001.

We calculated the average scores of two raters and system-wise scores, and overall scores are shown in Table 1. In the majority of the physiological systems, there was a difference in scores among the LLMs with an overall performance highest for ChatGPT and lowest for Bing.

**Table 1:** Domain-wise scores of ChatGPT, Bing, and Bard in solving physiology cases

Domain in physiology	ChatGPT	Bing	Bard	p-value
General (n=2)	3.25±0.35	2.5±0.35	2.63±0.53	0.67
Nerve-muscle (n=2)	3.5±0	2.75±0.35	3.5±0	0.33
Central nervous system (n=8)	3.22±0.24	2.53±0.3	3.16±0.31	0.0007
Cardiovascular (n=11)	3.09±0.35	2.25±0.56	2.82±0.55	0.0005
Blood and immunity (n=10)	3.28±0.29	2.08±0.79	2.88±0.46	<0.0001
Gastrointestinal (n=9)	3.25±0.25	2.58±0.39	2.91±0.31	0.0007
Renal (n=7)	3.14±0.56	1.64±0.48	2.89±0.19	0.0009
Temperature (n=2)	3.5±0	1.5±0.71	3.25±0.35	0.33
Respiratory (n=7)	3.14±0.24	1.57±0.35	2.79±0.39	0.0003
Special sense (n=5)	3.2±0.27	2±0.35	3.1±0.22	0.12
Endocrine (n=5)	3.1±0.22	1.9±0.22	3±0	0.12
Reproductive (n=9)	3.17±0.25	2.26±0.65	2.61±1.02	0.015
Overall (n=77)	3.19±0.3	2.15±0.6	2.91±0.5	<0.0001

The average ICC values for ChatGPT, Bing, and Bard were 0.858 (95% CI: 0.777 to 0.91, p<0.0001), 0.975 (95% CI: 0.961 to 0.984, p<0.0001), and 0.964 (95% CI: 0.944 to 0.977, p<0.0001), respectively. These ICC values indicate a stronger inter-rater agreement level of agreement between the raters for each language model's performance.

## Discussion

We found that ChatGPT consistently achieved the highest scores among the three LLMs, while Bing consistently obtained the lowest scores. This observation was confirmed by both the first and second raters. Furthermore, an average of the scores given by the two raters followed a consistent pattern, where ChatGPT outperformed Bing and Bard across the majority of physiological systems.

A study by Rahsepar et al. showed that ChatGPT exhibited higher accuracy compared to Google Bard in answering common lung cancer questions [7]. In contrast, a study by Raimondi showed different results. They found that, in the Royal College of Ophthalmologists fellowship exams, Bing Chat performed the best among the three AI systems, while ChatGPT had the lowest accuracy [8]. A study by Ali found that ChatGPT performs better than Bard in answering higher-order knowledge questions in neurosurgery oral board preparation questions [9]. Hence, the performance may vary according to various domains of the medical field.

Several potential underlying reasons may contribute to these performance differences. One plausible factor could be the varying capabilities and design of each language model. We presume that ChatGPT might have undergone more advanced training algorithms, received higher-quality training data, or been fine-tuned more effectively for physiology-related tasks. Additionally, ChatGPT's contextual understanding and coherence in generating responses may have played a role in earning higher scores. Moreover, the expertise and potential biases of the raters themselves could have influenced the evaluations. It is also essential to consider the model's handling of uncertainty and consistency in responses as contributing factors. Further research and investigation are required to comprehensively understand the nuanced reasons behind the observed performance disparities among the language models [10].

The use of LLMs in medical education has shown considerable promise in transforming traditional learning methodologies. LLMs, such as ChatGPT, have demonstrated their ability to process vast amounts of medical literature and provide contextually relevant information, making them valuable resources for both educators and students [11-14]. By leveraging LLMs, medical educators can offer interactive and dynamic learning experiences, allowing students to access up-to-date medical information, review complex concepts, and engage in problem-solving scenarios. Moreover, LLMs can enhance the efficiency of knowledge retrieval, providing quick answers to medical queries and supporting evidence-based decision-making. Integrating LLMs into medical education can foster self-directed learning, critical thinking, and analytical skills, empowering the next generation of healthcare professionals with cutting-edge resources and fostering continuous professional development in the rapidly evolving medical field [15]. However, careful consideration of the limitations and potential biases of LLMs is essential to ensure their responsible and ethical use, as well as to complement their role with hands-on, practical training and mentorship in clinical settings [16].

The study has some limitations. The study focused on a specific set of LLMs, namely, ChatGPT, Bard, and Bing, which may not represent the entire spectrum of LLMs available. Including a broader range of LLMs could provide a more comprehensive understanding of their capabilities in this context. Additionally, the study's assessment of LLM performance was based on responses to pre-defined case vignettes, limiting the exploration of their adaptability to a wider variety of clinical scenarios. There may be chances of bias due to its training data. Moreover, the study relied on the ratings of two physiologists, which, although valuable, may still introduce subjectivity and inter-rater variability in the evaluation process. Furthermore, the study's findings may not be generalizable to other medical specialties, as the efficacy of LLMs could vary depending on the complexity and domain-specific nature of different medical fields. Lastly, the study's design did not explore the potential biases or limitations of LLMs in their responses, which could be crucial in real-world applications.

## Conclusions

This study provides valuable insights into the application of LLMs in solving physiological case vignettes in medical education. The findings demonstrate the potential of LLMs, particularly ChatGPT, in offering accurate and contextually relevant responses to complex medical scenarios in physiology. Further research should explore a wider range of LLMs, examine adaptability to diverse clinical scenarios, and address potential biases in LLM responses.
